# Rheumatic Heart Disease among Pregnant Women with Cardiac Diseases in a Tertiary Care Center of Nepal: A Descriptive Cross-sectional Study

**DOI:** 10.31729/jnma.6177

**Published:** 2021-05-31

**Authors:** Basant Sharma, Eliza Koirala, Sudhir Regmi, Jaya Dhungana, Bandana Khanal Neupane, Suraj Bhattarai

**Affiliations:** 1 Department of Obstetrics and Gynecology, Chitwan Medical College, Bharatpur, Nepal; 2 Department of Anesthesia and Critical Care, Chitwan Medical College, Bharatpur, Nepal; 3 Department of Cardiology, Chitwan Medical College, Bharatpur, Nepal; 4 School of Nursing, Chitwan Medical College, Bharatpur, Nepal; 5 Department of Global Health, Global Institute for Interdisciplinary Studies, Kathmandu, Nepal

**Keywords:** *cardiac disease*, *Nepal*, *pregnancy*, *rheumatic heart disease*

## Abstract

**Introduction:**

Cardiac disease in pregnancy is a major cause of maternal mortality and morbidity in women, particularly in resource-limited countries like Nepal. Rheumatic Heart Disease is the commonest cardiac disease complicating pregnancy. There is very limited data and evidence from Nepal regarding rheumatic heart disease complicating the pregnancy. The study aims to find out the prevalence of rheumatic heart disease among cardiac disease patients in a tertiary care hospital.

**Methods:**

A descriptive cross-sectional study was conducted among 41 women with cardiac disease who delivered babies at Chitwan Medical College from 1st January 2018 to 31st December 2019, after taking ethical approval from the Institutional Review Committee. A convenient sampling method was used. Statistical Package for the Social Sciences was used for data analysis. Point estimate at 95% Confidence Interval was calculated along with frequency and proportion for binary data.

**Results:**

Among 41 pregnant women with cardiac disease, 32 (78%) (95% Confidence Interval = 65.32-90.68) had rheumatic heart disease. The mean age of the affected pregnant women was 24.9±4.49 years. Out of 32 patients with rheumatic heart disease, postpartum haemorrhage was the most common maternal complication 5 (15.6%) followed by hypertension 4 (9.7%).

**Conclusions:**

Rheumatic Heart Disease was highly common among pregnant women with cardiac disease.

## INTRODUCTION

Cardiac disease in pregnancy is a high-risk condition and a major cause of maternal mortality and morbidity, particularly in South Asia.^1^ Although direct or immediate death due to cardiovascular disease is rare, it is an important indirect cause of maternal death worldwide, with an attributable rate of two deaths per 100,000 pregnancies.^2^

Cardiovascular physiological changes during pregnancy impose an additional load on the cardiovascular system of women with underlying heart disease which increases morbidity and mortality during pregnancy and at the time of delivery.^3^ Among the cardiac disease, Rheumatic Heart Disease is the commonest cardiac disease complicating pregnancy.

There is very limited data and evidence from Nepal regarding the rheumatic heart disease among cardiac disease patients. The study aims to find out the prevalence of rheumatic heart disease among cardiac disease patients in a tertiary care hospital.

## METHODS

We conducted a descriptive cross-sectional study among cardiac disease women who delivered at Chitwan Medical College from 1st January 2018 to 31st December 2019. Ethical approval was taken from the Institutional Review Committee of Chitwan Medical College (IRC Reference number: CMC-IRC/076-077-130). All women with known or newly diagnosed heart disease admitted to the hospital for safe confinement of delivery and women who presented with cardiac problems resulting in delivery, during the period were included in the study. Women who were admitted to the hospital for termination of pregnancy and those admitted to the cardiology department for exacerbations of cardiac symptoms but not resulting in delivery were excluded from the study. A convenient sampling method was used.

The sample size was calculated by using formula,


$$\begin{array}{ll} \text{n} & \text{= }{\text{Z}}^{\text{2}}\times \text{p}\times q\;/{\text{e}}^{\text{2}}\\ & \text{= }{\left(1.96\right)}^{\text{2}}\times (0.89)\times (0.11)/{(\text{0.1)}}^{\text{2}}\\ & \text{= }37.6\\ & =38 \end{array}$$

Where,

n = minimum required sample size
Z = 1.96 at 95% Confidence Interval
p = prevalence, 89%^7^
q = 1-p
e = margin of error, 10%

The calculated sample size is 38. Taking a non-response rate 7%, the sample size is 41.

We recorded socio-demographic information, maternal characteristics, New York Heart Association (NYHA) functional class, valvular pathologies by echocardiography, delivery methods and obstetric outcomes.^4^

All pregnant women with cardiac disease received extended care because of associated complications. Additional investigations (other than routine antenatal investigations) included electrocardiography (ECG), and echocardiography. Vaginal delivery was prioritized for each case unless overridden by obstetric indications.

The maternal outcome was recorded in terms of termination of medical complications, critical care need, and mortality. The fetal outcome was recorded in terms of a 5-minute Apgar score, prematurity, neonatal intensive care (NICU) admission, and mortality.^5^

All personal identifiers of the patients were removed while transferring primary data into the data management software for data analysis purpose, so informed written consent was not required from the patients.

Statistical Package for Social Sciences (SPSS) 21.0 version was used for all data analyses. Categorical data were expressed as frequencies whereas continuous variables were presented as mean±SD or median whichever appropriate. Point estimate at 95% Confidence Interval was calculated along with frequency and proportion for binary data.

## RESULTS

Among 41 women pregnant with cardiac disease delivering baby at hospital, 32 (78%) (95% Confidence Interval = 65.3290.68) women had rheumatic heart disease. The mean age of the affected mothers was 24.9±4.49 years. Out of 32 mothers with RHD, 16 (50%) had isolated valvular heart disease, mitral stenosis being the commonest. Regarding the severity of diseases (Table 1), there were almost an equal proportion of mild, moderate and severe valvular lesions.

**Table 1:** Severity of rheumatic heart diseases in pregnant women (n = 41).

Characteristics	n (%)
Rheumatic heart disease	32 (100)
Mild	10 (31.25)
Moderate	12 (37.5)
Severe	10 (31.25)

Among 41 women with cardiac disease, 19 (46.3%) were primigravida, 18 (43.9%) were the second gravida and 4 (9.7%) were multigravida. Only 14 (34%) were booked cases. The majority of 33 (80.4%) mothers had a term delivery whereas 8 (19%) were preterm with no post-term delivery.

Among total study population, most of the mothers 32 (78%) underwent caesarean delivery, while the remaining 9 (22.0%) underwent normal vaginal delivery. Among the mother with rheumatic heart disease, the indication for operational delivery was cardiac condition of mother 10 (31.2%), previous C-section 6 (18.7%), fetal malpresentation 5 (15.6%), fetal distress 4 (12.5%) and others 7 (21.8%).

Out of the 32 patients with rheumatic heart disease, according to New York Heart Association (NYHA) functional classification, most of the patients 15 (46.9%) were in grade I followed by 11 (34.4%) patients in grade II while 1 (3.1%) in grade IV (Table 2).

**Table 2:** New York Heart Association (NYHA) Functional Classification grading in patients with rheumatic heart disease (n = 32).

Outcome indicators	RHD cases n (%)
^*^NYHA grade	
Grade I	15 (46.9)
Grade II	11 (34.4)
Grade III	5 (15.6)
Grade IV	1 (3.1)

* New York Heart Association (NYHA) is a functional classification of the patient based on symptom severity (degree of limitation of physical activity) and the amount of time or activities required to provoke symptoms (fatigue, palpitation or dyspnea). Grade I means normal cardiac function and Grade IV signifies severe cardiac disease.^4^

Among them, postpartum haemorrhage was the most common maternal complication 5 (15.6%) followed by hypertension 4 (9.7%) (Table 3). Seventeen (53.1%) mothers were admitted to the coronary care unit due to cardiac issues. Preterm birth 6 (14.6%) was the most common neonatal complications followed by birth asphyxia 4 (9.7%) then meconium aspiration 2 (4.8%) in mothers with rheumatic heart disease.

**Table 3:** Obstetric outcomes of women with rheumatic heart disease (n = 32).

Maternal outcome	n (%)
Any maternal complication	13 (40.6)
Postpartum hemorrhage	5 (15.6)
Hypertension	4 ( 9.7)
Hypothyroidism	1 ( 2.4)
Congestive cardiac failure	2 (4.8)
Pulmonary embolism	1 (2.4)
CCU admission	17 (53.1)
Median CCU stay in days (range)	3 (1 to 6)
Maternal death	1 (3.1)
Fetal outcome	
5-min ^**^Apgar score less than 7	11 (34.4)
Any fetal complication	12 (37.5)
Birth asphyxia	4 (9.7)
Meconium aspiration	2 (4.8)
Preterm birth	6 (14.6)
NICU admission	13 (40.6)
Neonatal death	3 (9.4)

** Apgar is a standardized clinical assessment of newborns consisting of 5 components (heart rate, respiratory effort, muscle tone, reflex irritability, colour), each of which is given a score of 0, 1, or 2. Apgar score of 7-10 at 5 minutes after birth means normal child, however, the score of fewer than 7 warrants repeat assessments every 5 minutes up to 20 minutes. A persistent low Apgar score signifies a depressed or non-reassuring baby.^5^

Fourteen out of 41 (34.1%) cases in our study had a postcardiac surgery status at the time of pregnancy. The principal valve involved was the mitral valve. Seven (17.07%) patients underwent mitral valvotomy, and 2 (4.87%) cases had a mitral valve replacement. Three (7.31%) cases had had ASD closure and two (4.8%) had VSD closure. Those women with mechanical valve replacement received anticoagulants either heparin (low molecular weight or unfractionated) or warfarin, depending on their gestational period, labour or postpartum status, as per protocol.

## DISCUSSION

Cardiac disease in pregnancy is a common cause of maternal and perinatal morbidity and mortality; particularly in developing countries.^6^ Similar to the study by Chhetri S, et al. our study showed Rheumatic heart disease as the commonest cardiac disease complicating pregnancy.^7^ Our study reported no stillbirths in comparison to 4 (9%) cases of stillbirths in the latter.^7^ The difference could be due to the effective implementation of the government's Safe Motherhood programme which has encouraged more antenatal visits resulting in early diagnosis and management of various cardiac and obstetric conditions.^8^

The cardiac lesions associated with cardiac disease in pregnancy vary with the study population and study setting. Only a portion of them is noticed during pregnancy or delivery.^9^ In our study, rheumatic heart disease (RHD) was reported four times more frequently than other heart disease. This finding is in contrast with a study from Canada that reported the majority (81.4%) of maternal cardiac disease to be congenital.^10^ While RHD incidence has been greatly reduced in developed countries due to widespread use of effective antibiotics against Group A streptococcal infections, its high prevalence in our setting indirectly indicates sheer negligence of streptococcal infections (throat, skin) among adolescent and young girls.

The RHD prevalence in our setting was 5.8 per 1000 pregnancies, which is comparable to other South Asian countries.^6,7^ According to the studies, it complicated about 0.3% to 3.5% of women in childbearing age, and accounted for about 30% of cardiac diseases in pregnancy in the developed and as high as 90 % in the developing countries.^11-13^ Among pregnant women with RHD, mitral stenosis was found to be the predominant valvular lesion followed by mitral regurgitation. Ten (31.2%) mothers with RHD has severe mitral stenosis which comparable with a study done in the eastern region of the country where it is 31%.^7^

Out of 32 pregnant women with rheumatic heart disease, 11 (34.3%) were primigravida, which is quiet less to the findings from another tertiary centre (67%) in Nepal.^7^ Although a majority of women had term delivery, the cesarean section rate was too high 32 (78%) at our centre, compared to other South Asian countries.^6,7,11^ The high rate of CS delivery was due to the high proportion of women with severe cardiac condition (31.2%), previous cesarean section ( 18.7% ) as well as the high rate of fetal complications (39%). Another reason for the high operational delivery rate could be the lack of a monitoring facility at our centre for conducting vaginal birth after cesarean (VBAC) or trial of labour after cesarean (TLOC).

Six maternal deaths (0.001%) were reported out of 5478 deliveries conducted at our centre during the study period, out of which only one was attributed to rheumatic heart disease. Our finding is supported by a study conducted in another centre of Nepal, although the study was conducted in relatively fewer patients of which only a few were cardio-symptomatic.^7^ About 53% of mothers with RHD required admission to the CCU and 39% of neonates from RHD mothers were admitted to the NICU. NYHA grade III or IV, presence of pulmonary hypertension and severe mitral stenosis are the predictors of maternal and perinatal deaths.^14^ An analysis from a prospective multi-centric study showed that prior cardiac events or arrhythmia, NYHA functional class II or above, left heart obstruction and systemic ventricular dysfunction could be the predictors of adverse cardiac events during pregnancy, whereas maternal mitral stenosis, smoking, multiple gestations and use of anticoagulants as the predictors of neonatal events.^9,15-16^ But a majority of mothers in our study 26 (81%) with RHD has NHYA I, II which are good predictors. There were three (7.3%) perinatal deaths reported in our study as described in Table 2, and the factors associated were birth asphyxia, meconium aspiration syndrome, and preterm birth.

Our study has few significant limitations. Due to the small sample size, the correlation of risk factor with obstetric outcomes could not be analyzed as it is a descriptive crosssectional study. Also, since we included only cases that delivered, the study missed hospital admissions for termination of pregnancy and admissions in the cardiology department for exacerbations of cardiac symptoms not resulting in delivery. Also, our study did not address the late perinatal complications that could have happened after discharge from the hospital. The absence of pediatric echocardiography at our setting prohibited complete cardiac evaluation of the neonates for inherited congenital heart disease.

## CONCLUSIONS

Rheumatic heart disease is highly common among the pregnant women with cardiac disease delivering at our centre. Postpartum haemorrhage was the most frequent complication in mothers. Larger studies with longer prospective follow up with systematic screening for cardiac disease in the early trimester, with documentation of early hospital admissions for cardiac and obstetric issues will provide robust data on the burden of cardiac disease complicating pregnancy.
